# Radiographic progression in early rheumatoid arthritis patients following initial combination versus step-up treat-to-target therapy in daily clinical practice: results from the DREAM registry

**DOI:** 10.1186/s41927-018-0009-8

**Published:** 2018-01-17

**Authors:** Laura M. M. Steunebrink, Letty G. A. Versteeg, Harald E. Vonkeman, Peter M. ten Klooster, Monique Hoekstra, Mart A. F. J. van de Laar

**Affiliations:** 1 Arthritis Center Twente, Department of Rheumatology, Medisch Spectrum Twente, PO BOX 50 000, 7500 KA Enschede, The Netherlands; 2 Department of Psychology, Health & Technology, University of Twente, Enschede, The Netherlands; 3 Department of Rheumatology, Isala, Zwolle, The Netherlands

**Keywords:** Early rheumatoid arthritis, Treat-to-target (T2 T), Remission, Radiographic progression, Joint damage

## Abstract

**Background:**

Early and intensive targeted treatment with disease modifying anti-rheumatic drugs (DMARDs) has been shown to lead to substantial reductions in disease activity and radiograph damage in patients with early rheumatoid arthritis (RA). The aim of this quasi-experimental study was to compare the first-year radiographic progression rates between a treat-to-target (T2 T) strategy with initial combination therapy (strategy II, started in 2012) versus an initial step-up monotherapy (strategy I, started in 2006).

**Methods:**

A total of 128 patients from strategy II was individually matched with 128 patients from strategy I on sex, age (± 5 yrs.) and baseline disease activity (± 0.5 on the DAS28). Differences in radiographic progression (Sharp/van der Heijde) scores (SHS) and the number of patients experiencing a minimal clinically important difference (MCID; ≥ 5 SHS points) between both strategies were tested with Mann Whitney U and chi-square tests. Next, linear and logistic regression analyses were performed to examine which baseline variables were associated with radiographic progression scores and the probability of experiencing an MCID within 1 year.

**Results:**

Patients with initial combination therapy had slightly higher baseline disease activity scores and pain scores, but better mental health scores. Patients with initial monotherapy had significantly more, and more frequently clinically relevant, radiographic progression after 1 year. Experiencing a MCID was independently associated with fewer tender joints (*p* = 0.050) and higher erythrocyte sedimentation rate (*p* = 0.015) at baseline.

**Conclusion:**

Treating early RA patients with initial combination therapy results in better radiographic outcomes than initial monotherapy in daily clinical practice.

**Trial registration:**

Netherlands Trial Register NTR578, 12 January 2006.

## Background

Rheumatoid arthritis (RA) is characterized by joint inflammation leading to joint destruction and related to a decrease in functional capacity, work disability, and reduced quality of life [1]. Prevention of structural damage is an important goal in the treatment of RA. In the last years, research has shown that early intensive treatment improves both the short- and long-term outcomes of RA [2–6]. The benefits of early use of (combinations of) disease modifying anti-rheumatic drugs (DMARDs) and biological agents [7–12], in combination with protocolled treatment aimed at a predefined goal (treat-to-target (T2T)) [13–15], has led to a change in traditional treatment paradigms. More specifically, early and intensive targeted treatment with DMARDs has been shown to lead to substantial reductions in disease activity [5, 6] and radiologic damage in patients with early RA [8, 13, 16–21].

However, in some early RA patients joint damage progresses even during DMARD use. In fact, even if DMARDs are initiated ‘very early’ in the disease, some patients may still develop erosions and progressive joint damage [2, 4, 22, 23]. Previous research has shown that in 7–17% of patients with RA in prolonged clinical remission, progression of joint damage still occurs [24, 25]. Van der Kooij et al. (2009) showed that 10–33% of patients in drug-free remission still showed progressive joint damage over a period of 4 years follow-up [26]. Whether the complete absence of arthritis activity prevents further joint damage in all patients, is still a matter of debate.

Previously, we demonstrated that implementation of a step-up T2T strategy in RA in daily clinical practice led to limited radiographic damage during a follow-up of 3 years [27, 28]. We also showed that while a T2T strategy with initial combination therapy was not superior to a T2T strategy with step-up therapy in the proportion of patients in remission at 12 months follow-up (77% versus 72%, respectively), the strategy with initial combination therapy did result in a significantly shorter time until remission. At 6 months, mean disease activity scores were lower in patients with initial combination therapy than in those with step-up therapy [6]. This is in line with clinical trials showing that initial combination therapy results in more rapid improvements in disease activity, daily functioning and quality of life than initial monotherapy [9, 13, 29, 30]. However, whether initial combination therapy and the subsequent shorter time to remission, as compared to initial step-up monotherapy, also results in better radiological outcomes has not been well studied in real-life clinical practice. Therefore, the aim of the present study was to compare the first-year radiological progression rates between a T2T strategy with initial combination therapy versus a T2T strategy with initial step-up monotherapy within the Dutch RhEumatoid Arthritis Monitoring (DREAM) registry.

## Methods

### Data selection and study design

This study used data from the ongoing DREAM T2T remission induction strategies I (initial step-up monotherapy) and II (initial combination therapy), two observational, multicenter strategies which were established in 2006 and 2012, respectively [5, 6, 27]. In both strategies, adults ≥18 years with a clinical diagnosis of RA and a disease duration (time from the diagnosis to the start of therapy) < 1 year were enrolled consecutively immediately after a clinical diagnosis of RA. For this study, data were used from two participating hospitals; Medisch Spectrum Twente in Enschede and Isala in Zwolle, both in The Netherlands. Patients included from 2006 to 2012 were used for strategy I, and patients included from 2012 to 2013 were used for strategy II. Both treatment strategies were in line with clinical practice and comply with current guidelines for treatment of RA. Exclusion criteria for both strategies were use of prednisolone ≥10 mg/day or previous or current treatment with disease-modifying antirheumatic drugs (DMARDs). The Medical Ethics Committees of the Medisch Spectrum Twente, Enschede and Isala, Zwolle hospitals determined, in accordance with Dutch Law on medical-scientific research with humans, that no ethical approval was required because all data were collected in the course of regular daily clinical practice. Nonetheless, patients were completely informed and informed consent was obtained from each patient.

At the time of the current analysis, 137 patients had a follow-up of at least 1 year in strategy II. For the aim of this quasi-experimental study, a total of 128 patients from strategy II could be individually matched with 128 patients from strategy I on sex, age (± 5 yrs.) and baseline disease activity (± 0.5 on the DAS28).

### Treat to target protocol

Patients in both strategies were treated according to a T2T strategy with protocolized treatment adjustments aiming at remission (DAS28 < 2.6), details of which have previously been published [5, 6, 27]. Briefly, the main differences between both strategies were; time moments of evaluation, and the medication that was started immediately after diagnosis (mono/step-up therapy versus combination therapy). In strategy I, patients were evaluated at 0, 8, 12, 20, 24, 36, and 52 weeks and every 3 months thereafter. In strategy II, patients were evaluated at months 0, 2, 4, 6 and every 3 months thereafter.

In strategy I, treatment protocol was an initial monotherapy of 15 mg/week methotrexate (MTX), with folic acid taken at the second day after MTX. In case of insufficient response (DAS28 ≥ 2.6) at the subsequent time-points, the following per protocol treatment steps were advised: after 2 months, MTX dosage was increased to 25 mg/week; after 3 months sulfasalazine (SSZ) 2000 mg/day was added; in week 20 SSZ dosages was increased to 3000 mg/day. Tumor necrosis factor inhibitor (TNFi) was prescribed at week 24 for patients with persistent moderate disease activity (DAS28 remained ≥3.2). If remission is achieved with DMARDs and/or TNFi, while maintaining remission for at least 6 months, medications were tapered and if possible discontinued starting with the TNFi and subsequently with the DMARDs.

In strategy II, treatment protocol was an initial combination therapy of MTX 20 mg/week and hydroxychloroquine (HCQ) 200 mg twice daily. As bridging therapy, an optional intramuscular triamcinolone injection to a maximum dosage of 120 mg could be given. After 1 month, MTX dosage was increased to 25 mg/week, independent of disease activity. After 2 months, in case of persistent disease activity (DAS28 ≥ 2.6), MTX dosage was further increased to 30 mg/week and an extra optional intramuscular triamcinolone injection could be administered. TNFi was prescribed at 4 months for patients with persistent moderate disease activity (DAS28 ≥ 3.2). If sustained remission for at least 6 months remission was achieved with DMARDs and/or TNFi, medications were tapered and if possible discontinued starting with the TNFi and subsequently with the DMARDs.

### Assessments

At each assessment, data were collected on various clinical and patient-reported outcome measures, including measures of disease activity, health related quality of life, physical functioning, and laboratory measures. Disease activity was assessed by trained rheumatology nurses using the Disease Activity Score for 28 joints (DAS28), consisting of a 28 swollen and tender joint count, the erythrocyte sedimentation rate (ESR) and a 100 mm visual analog scale (VAS) on general health (“Considering all the ways your arthritis affects you, how are you doing now?”, where 0 = “very good” and 100 = “very bad”) [31]. The Health Assessment Questionnaire Disability Index (HAQ-DI) was used to assess physical function [32]. Furthermore, patients rated their pain in the past week on a 100 mm VAS (0 = “no pain” and 100 = “unbearable pain”) and completed the Short Form Health Survey with 36 items (SF-36) in order to assess their current physical and mental health status [33]. Radiographs of hands and feet were obtained at baseline and annually thereafter. Radiographs were evaluated by two trained readers together, according to the modified Sharp/van der Heijde score (SHS) method [34], and a consensus score was obtained. Readers were not blinded to treatment strategy or assessment time point of the radiographs. A patient was classified as having erosive disease if the Sharp/van der Heijde erosion score was ≥1. Clinically relevant radiographic progression (minimal clinically important difference; MCID) was defined as an increase of ≥5 in the total SHS score [34].

### Statistical analysis

Descriptive statistics for categorical and continuous variables were reported as frequencies, percentages, means and standard deviations (SD). If continuous variables were not normally distributed, the median with the corresponding interquartile range (IQR) was reported. To test for any baseline differences between both strategies, we performed independent t-tests for normally distributed variables, Mann-Whitney U tests for non-normally distributed variables and chi-square tests for categorical variables. As only two time points were examined (baseline and 12 months), radiographic progression was calculated for observed values only. Differences between both strategies in one-year radiographic progression and the proportion of patients experiencing an increase of ≥5 SHS points (MCID) were tested using Mann Whitney U test and chi-square test. Group differences in progression between strategies were additionally visualized with a cumulative probability plot [35]. Next, univariate and multivariate linear and logistic regression analyses were performed to examine which other baseline variables were associated with radiographic progression and experiencing MCID within 1 year and to test for possible interactions with treatment strategy. Continuous variables were mean centered to allow for meaningful interpretation of main effects in addition to the interaction. The linearity assumption of continuous variables in the linear regression analyses was checked with scatterplots. Variables significantly (*p* < 0.05) associated with progression or with a significant interaction term in univariate analysis were entered as a covariate into a multivariate linear and logistic regression analysis model. To avoid multicollinearity, Pearson correlations were calculated between the independent variables to check for multicollinearity problems (*r* > 0.5). The explained variance of the final linear and logistic models was examined using (Nagelkerke’s pseudo) *R*^2^. The final logistic model was additionally tested for goodness of fit using the Hosmer and Lemeshow test. All statistical calculations were performed using version 22 of the SPSS statistical package for Windows.

## Results

### Patient characteristics

A total of 256 patients was enrolled in the study, 128 patients for each T2T strategy. Baseline characteristic of patients in both strategies were generally similar (Table 1). Patients had active disease, with a mean DAS28 of 4.8 in the T2T strategy with initial combination therapy (strategy II) versus a mean DAS28 of 4.5 in the T2T strategy with initial monotherapy (strategy I). Most patients were female and the majority was anti-CCP positive. Patients starting with initial combination therapy had slightly higher baseline disease activity scores and pain scores, but better mental health scores. Patients receiving an injection of triamcinolone had higher baseline DAS28 scores than those that did not receive an injection in both strategy I (5.6 ± 1.5 versus 4.5 ± 1.1; *p* = 0.038) and strategy II (5.1 ± 1.1 versus 4.6 ± 0.9; *p* = 0.011). Approximately 18% of the patients received MTX at baseline subcutaneously, whereas 82% of the patients received MTX orally. At 12 months the majority of the patients in both strategies received conventional synthetic DMARDs (csDMARDs) only. In strategy I, almost 4% of the patients were prescribed a biological DMARD (bDMARD), versus almost 9% in strategy II. In strategy II there were slightly more patients in whom DMARD use was fully discontinued (8% versus 2%). Median dose of triamcinolone administered to patients at baseline in strategy I was 80 mg. 75% (3/4) of the patients received 80 mg triamcinolone and 25% (1/4) of the patients received 120 mg triamcinolone. The median dose of triamcinolone administered to patients at baseline in strategy II was 120 mg. 93% (62/67) of the patients received 120 mg triamcinolone and 7% (5/67) of the patients received 80 mg triamcinolone at baseline.

**Table 1:** Patient characteristics

Characteristics	Strategy I (*n* = 128)	Strategy II (*n* = 128)	*p*
Female, sex n (%)	79 (61.7%)	79 (61.7%)	1.000
Age, mean ± SD years	59.1 ± 13.0	59,5 ± 12.8	0.809
DAS28 - ESR, mean ± SD	4.5 ± 1.1	4.8 ± 1.1	0.026
ESR (mm/h), median (IQR)	22.0 (14.0–41.0)	29.0^a^ (14.0–45.0)	0.195
CRP (mg/l), median (IQR)	10.0^b^ (5.0–22.0)	11.5 (4.3–24.8)	0.786
Anti-CCP positive, n (%)	74^c^ (58.3%)	77^c^ (60.2%)	0.701
RF positive, n (%)	62 (48.4%)	76 (59.4%)	0.114
Number of SJC, median (IQR)	6.0 (3.0–9.0)	5.0 (2.0–10.0)	0.235
Number of TJC, median (IQR)	3.0 (1.0–7.0)	4.0 (2.0–10.0)	0.025
HAQ-SDI, median (IQR)	1.2^d^ (0.9–1.6)	1.0^e^ (0.4–1.5)	0.003
VAS well-being, median (IQR)	50.0 (28.3–65.0)	51.0 (35.0–70.0)	0.290
VAS pain, median (IQR)	50.0^f^ (39.8–64.0)	62.0^g^ (49.0–75.0)	0.001
SF36-PCS, mean ± SD	38.1 ± 7.6^h^	37.3 ± 9.2^i^	0.512
SF36-MCS, mean ± SD	40.7 ± 7.4^j^	44.9 ± 11.9^k^	0.003
BMI, kg/m^2^, mean ± SD	26.5 ± 4.8^l^	26.0 ± 4.1^m^	0.388
Baseline SHS-score, median (IQR)	3.0 (1.0–7.0)^n^	2.0 (1.0–5.8)^o^	0.119
Erosion, median (IQR)	0.0 (0.0–1.0)^n^	0.0 (0.0–1.0)^o^	0.012
Joint space narrowing, median (IQR)	2.0 (0.0–5.0)^n^	1.0 (0.0–1.0)^o^	0.586
Injection triamcinolone, n%	4 (3.1%)	67 (52.3%)	0.000

^a^(*n* = 127), ^b^(*n* = 121), ^c^(*n* = 127), ^d^(*n* = 102), ^e^(*n* = 87), ^f^(*n* = 122), ^g^(72), ^h^(*n* = 105), ^i^(*n* = 86), ^j^(*n* = 105), ^k^(*n* = 86),^l^(*n* = 126), ^m^(*n* = 119), ^n^(*n* = 126), ^o^(*n* = 124)

*DAS28* Disease Activity Score in 28 joints, *ESR* erythrocyte sedimentation rate, *CRP* C-reactive protein, *TJC* tender joint count, *SJC* swollen joint count, *HAQ-SDI* Health Assessment Questionnaire disability index (standard scoring), *SF-36* Short-Form 36 health survey (version 2), *PCS* physical component summary, *MCS* mental component summary, *BMI* body mass index, *RF* rheumatoid factor, *Anti-CCP* anti-cyclic citrullinated peptide

Within the first year, there were significantly more patients with registered complications in strategy II (*n* = 45 [35%]) than in strategy I (*n* = 15 [12%]; *p* < 0.01). In total, there were 27 complications registered in strategy I compared to 83 complications in strategy II. Complication in strategy I consisted of: malaise = 14; gastrointestinal = 2; lab abnormality = 4; skin/hair disorder/allergy = 3; infection = 2; cardiovascular event = 1; other = 1. Complication in strategy II consisted of: malaise = 38; gastrointestinal = 7; lab abnormality = 8; skin/hair disorder/allergy = 12; eye complaints = 8; pulmonary abnormality = 1; infection = 1; cardiovascular event = 2; other = 6.

### Radiographic progression

**Fig. 1:**
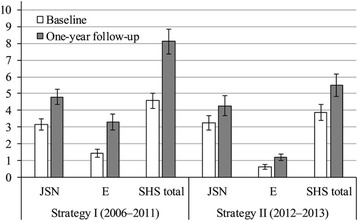
Radiographic damage in both strategies. The data are presented as the mean ± standard error of mean. JSN = joint space narrowing; E = Erosion; SHS = Sharp/van der Heijde score

Baseline radiographs were available for 250 patients (124 patients in strategy II and 126 patients in strategy I). One-year follow-up radiographs were available for 222 patients (104 patients in strategy II and 118 patients in strategy I). Baseline and follow-up SHS scores are presented separately for both treatment strategies in Fig. 1.

At baseline, median SHS scores were not significantly different (*p* = 0.119) between both strategies. Median baseline erosion scores tended to be slightly higher in strategy I (*p* = 0.012) with 43% (54/126) of the patients versus 30% (37/124) of the patients in strategy II having at least one erosion, while joint space narrowing (JSN) scores were not different between strategies with 71% (90/126) of the patients in strategy I versus 68% (84/124) of the patients in strategy II showing JSN.

At one-year follow-up, the median SHS score was significantly higher in patient treated with initial monotherapy (*p* = 0.001). The median SHS score increased from 3.0 (IQR 1.0–7.0) at baseline to 5.5 (IQR 3.0–12.0) in strategy I and from 2.0 (IQR 1.0–5.75) to 3.0 (IQR 1.0–9.0) in strategy II. One-year erosion scores were significantly different (*p* < 0.001) between both strategies, while JSN scores were not (*P* = 0.117). In strategy I, median erosion scores increased from 0.0 (IQR 0.0–1.0) to 2.0 (IQR 0.0–4.0) and median JSN scores increased from 2.0 (IQR 0.0–5.0) to 3.0 (IQR 1.0–7.0). In strategy II, median erosion scores increased from 0.0 (IQR 0.0–1.0) to 1.0 (0.0–2.0), while JSN scores increased from 1.0 (IQR 0.0–4.75) to 2.0 (IQR 1.0–6.0).

**Fig. 2:**
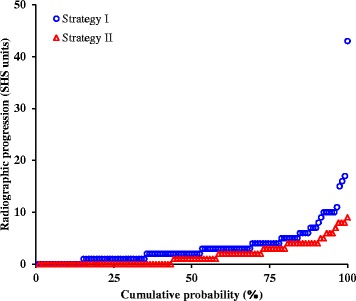
Cumulative probability of radiographic progression between both strategies

Median progression was significantly higher in strategy I (2.0; IQR 1.0–4.0) than in strategy II (1.0; IQR 0.0–3.0; *p* < 0.001). This difference was similar for both anti-CCP negative (2.0 [IQR 1.0–3.0] vs. 1.0 [IQR 0.0–2.0]; *p* = 0.023) and anti-CCP positive patients (3.0 [IQR 1.0–5.0] vs. 1.0 [QR 0.0–3.0]; *p* < 0.001). The difference in individual radiographic progression scores between both strategies was also visible in the cumulative probability plot (Fig. 2). Most notably, the proportion of patients with no radiographic progression at all was substantially higher strategy II (initial combination therapy) (43%) than in strategy I (initial monotherapy) (14%).

Also, significantly more patients treated with initial monotherapy had clinically relevant progression (≥ 5 SHS points) after 1 year (26/118 [20%]) than patients treated with initial combination therapy (10/104 [8%]; *p* = 0.012).

### Univariate associations with progression

Fewer tender joints (*p* = 0.033), higher ESR (*p* = 0.033), higher age (*p* = 0.042), and no triamcinolone injection (*p* = 0.007) were significantly associated with more radiographic progression within the first year in the total sample (Table 2). There were no significant interactions with strategy in the linear regression analyses. With respect to clinically relevant radiographic progression, fewer tender joints (*p* = 0.016), higher ESR (*p* = 0.038), positive anti-CCP (*p* = 0.040) and lower BMI (*p* = 0.039) were significantly associated with experiencing a MCID. Moreover, there was a significant strategy interaction with swollen joint count scores and SF-36 PCS scores. Swollen joint count scores were not associated with experiencing a MCID in strategy II (OR = 0.90; CI 95% 0.77–1.07; *p* = 0.227) but were positively associated in strategy I (OR = 1.10; CI 95% 1.00–1.22; *p* = 0.050). SF-36 PCS scores were not associated with reaching a MCID in strategy I (OR = 0.98 CI 95% 0.92–1.04 *p* = 0.503) but were positively associated with reaching a MCID in strategy II (OR = 1.11; CI 95% 1.00–1.22; *p* = 0.040).

**Table 2:** Univariate analyses of progression and minimal clinically important difference (MCID ≥ 5)

Variable	Progression			Minimal clinical important difference (MCID ≥5)		
	B	95% CI	*p*	OR	95% CI	*p*
Strategy (monotherapy)	1.79	0.75–2.82	0.001	2.66	1.21–5.82	0.015
TJC28	−0.11	−0.20– -0.01	0.033	0.89	0.81–0.98	0.016
SJC28	0.01	−0.10–0.13	0.805	1.03	0.96–1.10	0.471
ESR	0.03	0.00–0.05	0.033	1.02	1.00–1.03	0.038
CRP	0.02	−0.00–0.04	0.084	1.01	1.00–1.02	0.071
Wellbeing	−0.01	−0.04–0.01	0.196	0.99	0.98–1.01	0.319
PCS	0.01	−0.07–0.09	0.803	1.02	0.97–1.07	0.467
MCS	−0.03	−0.09–0.04	0.408	1.01	0.97–1 .05	0.653
RF	0.09	−0.45–0.62	0.755	0.78	0.54–1.14	0.202
Anti-CCP positive	0.86	−0.23–1.95	0.123	2.41	1.04–5.57	0.040
Age	0.04	0.00–0.08	0.042	1.02	0.99–1.05	0.251
Gender	0.28	−0.82–1.38	0.621	0.75	0.36–1.56	0.443
DAS28_ESR	−0.02	−0.51–0.47	0.948	0.94	0.67–1.31	0.708
Remission 52 weeks	−0.83	−2.07–0.41	0.188	0.64	0.29–1.42	0.272
Remission 6 months	−0.73	−1.79–0.34	0.179	0.54	0.26–1.11	0.094
Remission 3 months	−0.90	−1.99–0.19	0.104	0.71	0.33–1.54	0.388
HAQ-SDI	0.62	−0.46–1.69	0.260	1.00	0.54–1.85	0.994
BMI	−0.05	−0.17–0.07	0.417	0.91	0.83–1.00	0.039
Injection triamcinolone	−1.63	−2.81– -0.46	0.007	0.39	0.15–1.07	0.067

*TJC* tender joint count, *SJC* swollen joint count, *ESR* erythrocyte sedimentation rate, *CRP* C-reactive protein, *PCS* physical component summary, *MCS* Mental component summary, *DAS28* Disease Activity in 28 joints, *RF* rheumatoid factor, *Anti-CCP* anti-cyclic citrullinated peptide, *HAQ-SDI* Health Assessment Questionnaire disability index (standard scoring), *BMI* body mass index

### Multivariate analyses

Strategy I with initial monotherapy remained significantly associated with more radiographic progression and experiencing a MCID after controlling for covariates and strategy interactions in multivariate analyses (Tables 3 and 4). None of the covariates remained significantly associated with continuous radiographic progression scores (Table 3). For clinically relevant progression, fewer tender joints (*p* = 0.050) and higher ESR (*p* = 0.015) remained significantly associated with experiencing a MCID (Table 4).

**Table 3:** Multivariable linear regression analysis for progression

Variable	B	95% CI	*p*
Strategy (Initial monotherapy)	1.32	0.06–2.57	0.039
TJC	−0.08	−0.17 – 0.02	0.130
ESR	0.02	−0.00 – 0.05	0.074
Age	0.04	−0.01 – 0.08	0.089
Injection triamcinolone	−0.76	−2.19 – 0.68	0.298

R-square progression = 0.079; *N* = 121

All continuous variables were mean-centered to avoid multicollinearity

**Table 4:** Multivariable logistic regression analysis for clinically relevant progression (≥ MCID of 5)

Variable	OR	95% CI	*p*
Strategy (Initial monotherapy)	3.84	1.16–12.73	0.028
TJC	0.89	0.78–1.00	0.050
SJC	1.07	0.92–1.23	0.393
SJC x Strategy	1.17	0.90–1.51	0.236
ESR	1.03	1.01–1.05	0.015
Anti-CCP positive	1.91	0.66–5.48	0.229
SF-36 PCS	1.04	0.98–1.12	0.202
SF-36 PCS x Strategy	0.87	0.76–0.99	0.034
BMI	0.91	0.81–1.02	0.114

Nagelkerke R-square MCID = 0.266; *N* = 159

All continuous variables were mean-centered to avoid multicollinearity

*TJC* tender joint count, *SJC* swollen joint count, *ESR* erythrocyte sedimentation rate, *Anti-CCP* anti-cyclic citrullinated peptide

## Discussion

The aim of this study was to compare one-year radiographic outcomes of two treat-to-target strategies, with initial mono- versus combination therapy, in early RA patients in daily clinical practice. These two early RA strategies in the DREAM registry confirm that, overall, treat-to-target strategies result in limited short-term radiographic progression. We observed an even more favorable outcome among patients with early RA who were treated with initial combination therapy (strategy II), as compared to patients who were treated with initial monotherapy (strategy I). A substantially larger number of patients within strategy II showed no radiographic progression at all and only a small portion of patients showed clinically relevant progression.

Fewer painful joints and a higher erythrocyte sedimentation rate (ESR) at baseline turned out to be predictive of clinically relevant progression, independent of treatment strategy. Although high ESR is an established risk factor for progression [36–38], it was surprising that patients with fewer tender joints ended up with more structural progression. Although the exact reason for this finding is unknown, it could suggest that patients with a higher pain threshold may receive less than optimal treatment (e.g., less frequent glucocorticoid administrations or other treatment intensifications). Consequently, this finding deserves further study. Better physical health at baseline was predictive of clinically relevant progression in the strategy with initial combination therapy only. Treatment strategy remained the strongest independent predictor for the occurrence of radiographic progression after controlling for other potential predictors.

Previously, we demonstrated that patients treated according with initial combination therapy showed a more rapid improvement in disease activity than patients treated with step-up monotherapy [6]. Early and intensive treatment of RA is advocated, in order to prevent structural joint damage in the early phase of the disease and thereby prevent loss of function resulting from joint destruction and active arthritis [39]. Treatment with traditional DMARDs alone or in combination [9, 20] with glucocorticoids [40] has been shown to retard the progression of joint damage. In our study, the increase of median SHS score (progression) after 1 year of follow-up was significantly lower in patients who had been treated with initial combination therapy compared to patients who had been treated with initial step-up monotherapy. Our results are comparable to those of the FIN-RACO trial which showed that the short-term and long-term increase in median Larsen score was significantly lower in patients who were treated with combination DMARDs compared to patients receiving DMARD monotherapy during the first 2 years [41, 42]. Also, the COBRA study, although this study was not aiming at remission, compared step-down combination therapy with prednisolone, methotrexate, and sulfasalazine (SSZ) to SSZ monotherapy and showed that after 1 year the rate of progression of joint damage was lower in the combination therapy group and less persistent over 4–5 years of follow-up [20].

Similar results were demonstrated in the BeST study [21], where radiological results showed that patients who had been treated with initial combination therapy including prednisone had less progression of radiographic joint damage than patients treated with sequential mono-therapy. The BeST study also showed that the number of patients without any progression of radiographic joint damage was higher in the combination therapy group. In contrast to these studies, the tREACH trial found no difference in radiographic progression between initial triple DMARD therapy and intramuscular glucocorticosteroids versus initial triple DMARD therapy and oral glucocorticosteroids versus initial MTX monotherapy and oral glucocorticosteroids [43]. In the tREACH trial, all treatment groups used glucocorticoids, which might result in early control of the inflammatory disease, which in turn might lead to less short-term progression of damage. Our study in daily clinical practice confirmed that in early RA, starting with a combination therapy of multiple DMARDs has several positive outcomes. In general, it is assumed that rapid aggressive treatment may slow long-term progression [20]. From this perspective we might surmise that starting early RA treatment with a single DMARD would be a missed opportunity in a considerable number of patients.

The identification of possible prognostic factors of radiographic progression is relevant for tailoring treatment and for supporting the current treatment strategy. The strength and reliability of known prognostic factors may vary according to the outcome measure of interest. A systematic review by Carpenter (2016) indicated that RF, anti-CCP, along with increased markers of inflammation (ESR or CRP) were strongly associated with radiographic progression [44]. The ESPOIR study identified anti-CCP and baseline ESR as predictors of structural outcome [45]. Van der Heijde (1992) mentioned high disease activity measured as high ESR, CRP or DAS and a positive RF were all significantly associated with radiographic damage after 2 years of follow-up [46]. Our study cannot confirm all of these associations; but fewer tender joints and higher ESR were independently associated with radiographic progression in the total sample.

Glucocorticoids were part of strategy II, as bridging therapy, and were allowed in strategy I to reach remission. Glucocorticoids have previously been shown to retard radiographic progression [40, 47]. In the total sample, use of glucocorticoids at baseline was univariately associated with less radiographic progression. Looking at the initial combination strategy group only, however, there was no significant difference in radiographic progression between those patients who did or did not receive a baseline glucocorticoid injection (data not shown). Moreover, the association between corticosteroid use and radiographic progression did not remain significant in the multivariate model that included strategy group. Consequently, the current study, albeit not specifically designed to answer this question, could not confirm that glucocorticoids retard short-term radiographic progression. It is possible that this association is confounded by indication, as patients who received an injection of triamcinolone had higher baseline DAS28 scores in both strategies.

The major strength of this study is the use of real life data from consecutive patients, recently diagnosed with RA, who were being treated according to a state-of-the-art T2T remission induction protocol. Results from this study demonstrated similar or even better radiographic outcomes than several T2T RCTs. Since RCTs generally have more strict inclusion criteria and more controlled protocols, it is important that treatment outcomes are also examined in real-life settings. This study is one of the first to directly compare the radiographic outcomes of different T2T strategies in early RA patients in daily clinical practice. Another strength of the study is that the Sharp/van der Heijde method was used instead of the Larsen method for scoring radiographic progression. The Sharp van der Heijde index may be considered as the best tool for evaluating patients with early RA because of its sensitivity in detecting signs of early disease and the possibility of expressing anatomical damage progression quantitatively [48]. Although this study was not powered a-priori for the current comparison of radiographic outcomes, a post-hoc power analysis indicated that with the sample size of 128 patients per strategy we had >80% power to detect a small to moderate difference (d = 0.39) in progression between both strategies using a 2-sided Mann-Whitney u test with an alpha of 0.05.

The major limitation of this study is that it is a quasi-experimental study of two strategies separated over time. The first strategy started in 2006 with an initial step-up mono-therapy, the second strategy in 2012 started with an initial combination therapy. Also, both strategies differed not only with respect to initial DMARD therapy (step-up vs. combination), but also with respect to the use of glucocorticoids and MTX starting dose. Another limitation is the follow-up period of 1 year. Finally, an important limitation is that radiographs in both strategies were not scored in a randomized and blinded fashion, as is usually done in true clinical trials.

Although it is not a randomized trial, we still think that the design and results of the study allow us to compare between the two strategies. Both strategy cohorts consisted of very similar populations of all consecutive newly diagnosed RA patients, treated in the same hospitals by the same rheumatologists. Although early radiographic progression is strongly indicative of future radiographic progression, longer follow-up is needed to investigate whether initial combination therapy also shows long-term beneficial effects on radiographic progression. Long-term follow-up of the COBRA and FIN-RACO trials suggested a difference in progression of joint damage after 1–2 years between combination and monotherapy [49, 50], while the BeSt study did not after 1–5 years [51]. In the long-term follow-up of strategy I within the DREAM registry, patients with early joint damage demonstrated more continued radiographic progression [52]. Because patients in strategy II showed less early radiographic progression, initial combination therapy might also prevent the destruction of joints on the long-term.

## Conclusion

Patients treated with initial monotherapy had significantly more first-year radiographic progression than patients treated with initial combination therapy in daily clinical practice.

## Abbreviations

Anti-CCP: Anti-cyclic citrullinated peptide
BMI: Body mass index
CRP: C-reactive protein
DAS28: Disease activity score for 28 joints
DMARD: Disease modifying anti-rheumatic drug
DREAM: Dutch RhEumatoid Arthritis Monitoring registry
ESR: Erythrocyte sedimentation rate
HAQ: Health Assessment Questionnaire Disability Index
HCQ: Hydroxychloroquine
IQR: interquartile range
JSN: Joint space narrowing
MCID: Minimal clinically important difference
MCS: Mental component summary
MTX: Methotrexate
PCS: Physical component summary
RA: Rheumatoid arthritis
RF: Rheumatoid factor
SD: Standard deviations
SF36: Short Form Health Survey with 36 items
SHS: Sharp/van der Heijde score
SJC: Swollen joint count
SSZ: Sulfasalazine
T2 T: Treat-to-target
TJC: Tender joint count
TNFi: Tumor necrosis factor inhibitor
VAS: Visual analog scale
